# The Role of Fe, S, P, Ca, and Sr in Porous Skeletal Lesions: A Study on Non-adult Individuals Using pXRF

**DOI:** 10.1007/s12011-024-04187-4

**Published:** 2024-05-01

**Authors:** Ricardo A. M. P. Gomes, Lidia Catarino, Ana Luisa Santos

**Affiliations:** 1 Research Centre for Anthropology and Health (CIAS), Department of Life Sciences, University of Coimbra, Rua Do Arco da Traição, 3000-056 Coimbra, Portugal; 2 Carrera de Antropologia, University of Concepción, Barrio Universitário S/N, Concepción, Chile; 3 Geosciences Center, Department of Earth Sciences, University of Coimbra, Rua Sílvio Lima - Pólo II, 3030-790 Coimbra, Portugal

**Keywords:** Age at death, Anemia(s), Cribra, Spectroscopy, Trace elements

## Abstract

Portable X-ray fluorescence is a new tool in the study of human bone. This research aims to investigate if variations in bone elemental concentrations are related with porous skeletal lesions (PSLs). One hundred well-preserved non-adult skeletons aged 0–11 years were selected from the archaeological site Convent of São Domingos, Lisbon (18th–19th century). Measuring a standard reference material and calculating the technical error of measurement assured elemental data reliability. Moreover, measuring soil samples excluded possible contamination of bones with elements from the soil, except for Pb. Additionally, the Ca/P ratio indicates maintenance of bone integrity. Cribra cranii, orbitalia, humeralis, and femoralis were recorded as present/absent, and the estimated intra-/inter-observer errors were low. The multivariate analysis found higher odds of having cribra orbitalia (OR = 1.76; CI = 0.97–3.20) and cribra femoralis (OR = 1.42; CI = 0.73–2.74) in individuals with lower Fe and higher S. Furthermore, higher levels of P, Ca, and Sr increased the odds of individuals developing cribra femoralis (OR = 2.30; CI = 1.23–4.29). Age also correlated with increased odds of exhibiting cribra orbitalia (OR = 1.86; CI = 0.94–3.68), cribra femoralis (OR = 6.97; CI = 2.78–17.45), and cribra humeralis (OR = 8.32; CI = 2.71–25.60). These findings suggest a shared etiology for these three cribras, contrasting with the higher Fe levels in individuals with cribra cranii. Lower Fe and higher S levels in individuals with cribra suggest a complex etiology, possibly involving conditions like megaloblastic or chronic disease anemia(s). Age-related elemental changes support the hypothesis that age influences cribra frequencies. This study highlights PSL complexity and opens new avenues for research.

**Supplementary Information:**

The online version contains supplementary material available at 10.1007/s12011-024-04187-4.

## Introduction

Bone elemental concentration may offer a unique perspective in the study of human bones, since it allows to access possible homeostatic disruptions, based on chemical alterations detectable by spectroscopy [1, 2]. However, one of the main issues of spectrometric techniques is that they often require sample destruction [3]. The development of X-ray fluorescence, particularly with the recent versions of portable devices (pXRF), allows non-invasive procedures, which recently started to be used in osteological elemental studies [4–6].

The features of pXRF facilitate its application in the study of porous skeletal lesions (PSLs). This term has been used to encompass the set of porous alterations in specific regions of the skeleton: the cranial vault (cribra cranii), the orbital roof (cribra orbitalia), the neck of the humerus (cribra humeralis), and the femur (cribra femoralis). These lesions, commonly observed in the bioarchaeological record, are not only more frequent in non-adults, but also, according to some authors, do not develop in adulthood (e.g., 7–9). At the moment, PSLs are not described in the clinical literature, yet they have been investigated in the context of autopsies.

To the authors’ knowledge, the first investigation applying pXRF to the study of PSL was from Çirak [10]. The results were promising, as lower Fe levels were associated with cribra orbitalia, in two female adult skulls from Turkey (Byzantine period), yet the small sample size does not allow to draw definitive conclusions. More recently, Kilburn et al. [11] evaluated Ba, Sr, Pb, Zn, Fe, Mn, Ti, Ca, K, Al, P, Si, Cl, S, and Cu in 34 non-adults (humerus, femur, and tibia) from the UK (eighteenth–nineteenth century CE) and found no association between the calculated ratios and the presence of cribra orbitalia. Bone elemental concentration was also gauged through pXRF in individuals from the Coimbra Identified Skeletal Collections (Portugal) dated from 19th–20th century CE [12]. The authors did a comparative analysis between two groups: individuals with anemia as the cause of death (*n* = 19) and a control group (*n* = 26) characterized by causes of death unrelated to anemia. No statistical differences were found between these two groups, despite individuals with cribra cranii presenting higher levels of Fe and low levels of S. Lundová et al. [13] focused on the elemental concentrations of Ca, Fe, and Pb in 13 non-adults from 14th–16th century CE (Czechia) and found no significant differences between individuals with and without cribra orbitalia. Finally, the analysis of 107 non-adult individuals from two identified skeletal collections from Portugal (19–20 century CE) revealed that those individuals with cribra cranii presented higher levels of Fe and lower levels of S [14]. The authors proposed an association among sideroblastic anemia (SA) (related to increased Fe levels), poor nutritional status, and early alcohol consumption that led to the cribra expression. Additionally, concentrations of P, Ca, Sr, and Pb were significantly associated with increasing age at death, possibly reflecting normal skeletal growth.

As far as the authors know, these are the sole investigations to utilize pXRF in the study of PSL, with a focus on changes in Fe concentration. In fact, several bioarchaeological studies point to anemia as one of the main causes of cribra [15–18]. These arguments began to be defended as individuals with genetic hemolytic anemias presented severe skeletal lesions resulting from marrow hyperplasia [19, 20]. Given the increased need of oxygen in anemic states, the marrow may be stimulated to produce more or bigger red blood cells (RBCs), causing marrow expansion [21]. This, associated with erratic bone remolding, may lead to porous lesions [22]. As genetic hemolytic anemias are more specific to some global regions, it has been suggested that iron deficiency anemia (IDA), a more prevalent form, may better explain the widespread pattern of PSL [18, 23, 24]. Through the years, arguments have been raised in favor of [25, 26] and against [27–30] this possibility, and other forms of anemia (e.g., megaloblastic anemia, anemia of chronic disease) have been proposed. As mentioned by Brickley [31], the diagnosis of anemia in paleopathology must follow more rigorous criteria than just the observation of cribra. Furthermore, recent studies have opened a new path for the interpretation of PSL. Individuals with respiratory infections were found to have higher odds of exhibiting PSL, as observed in postmortem CT scans from non-adults [32, 33] and from identified skeletal collections [14, 34].

Various factors have been proposed as contributors to PSL, yet the etiological discussion remains multifaceted. Analyzing bone elemental concentration can deliver valuable insights to this complex framework. While the aforementioned studies focus solely on skeletal analysis, they underscore that soil characteristics, frequently altered by anthropic action, can induce post-depositional diagenetic changes. Thus, the main contribution of this study is to assess the reliability of elemental concentrations obtained through pXRF, accounting for potential soil influences, by comparing the elemental concentration of soil with the bone samples Additionally, it aims to contribute to the etiological discussion of PSL by reiterating differences in bone elemental concentration among non-adult individuals with or without cribra, already observed in previous studies.

The following hypotheses were established:

1. The comparison of bone and surrounding soil elemental concentrations enables the identification and/or exclusion of possible bone contamination by elements from the soil.
2. Bone elemental concentration varies with age, and in these non-adult bones, one is expected to find higher levels of P, Ca, and Sr in older individuals.
3. Higher concentrations of Fe and lower concentrations of S are anticipated in individuals with cribra cranii, similar to what was observed in non-adults and adults from the identified skeletal collections.

## Material and Methods

### The Individuals

**Fig. 1:**
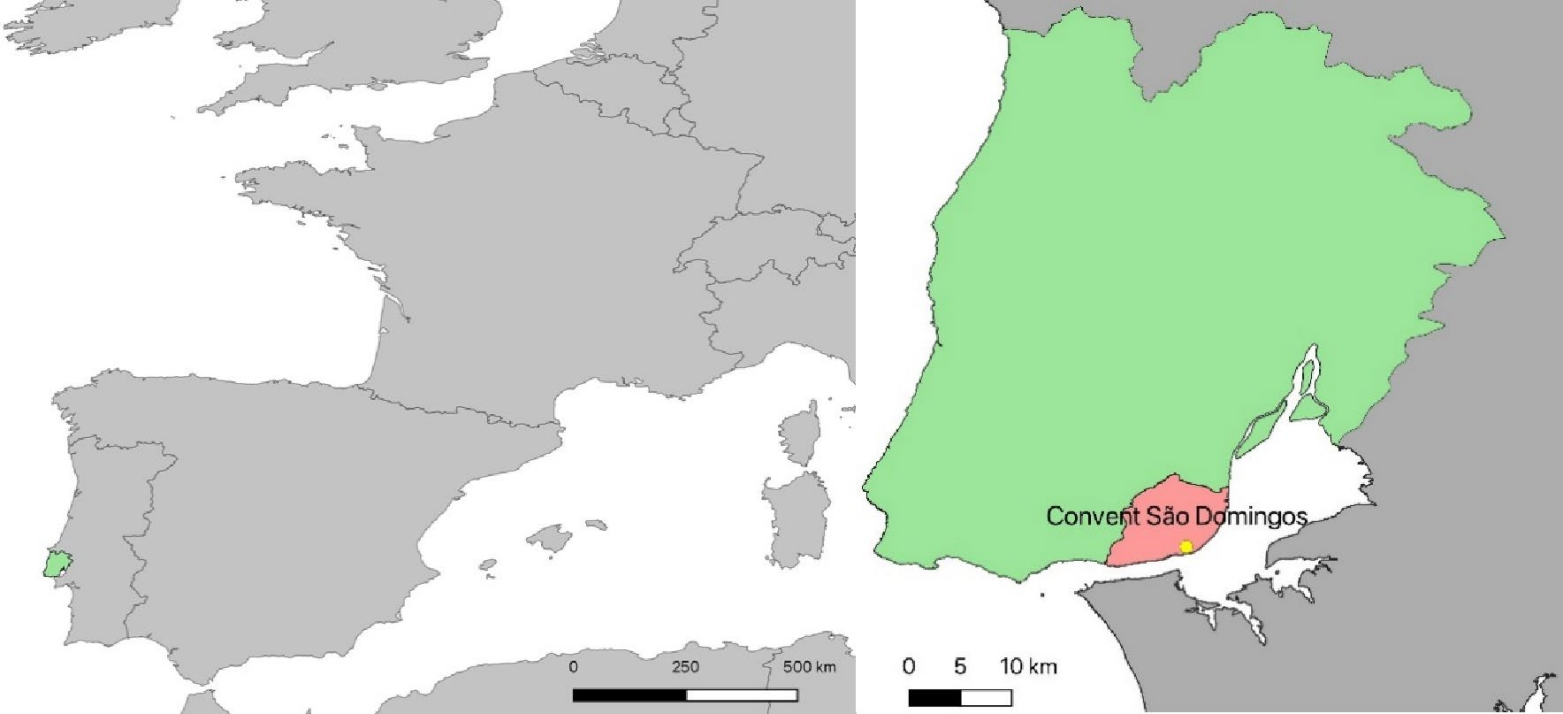
In green is the localization of Lisbon in Portugal (left), and in pink is the Lisbon district’s area with the exact location of the Convent São Domingos (right)

This research analyzed 100 non-adult individuals under the age of 11 years from the children’s necropolis of the Convent of São Domingos (SDC) in Lisbon. These individuals were exhumed following the archaeological work carried out by Era-Arqueologia SA between 2018 and 2023, during the reconstruction work to convert this building into a hotel. The SDC of the Dominican Order was built in the 13th century by royal initiative [35]. It is located in downtown Lisbon within a square with the same name (Fig. 1). Over the centuries, various events, such as the earthquakes of 1531 and 1755 and the fire of 1959, caused significant damage to its structure, leading to subsequent reconstructions [36]. After the expulsion of the religious orders in 1835, the building was sold with all its belongings, and the convent area was partially demolished, leaving only the church [37].

The SDC comprises two extensive necropolises: (1) the conventual, dated between the 16th century and the end of the 18th century, which extended along the corridor adjacent to the cloister and the convent’s old chapter room; and (2) the children’s necropolis, dated between the 18th century and the beginning of the 19th century, which occupied the entire cloister [38].

This research focuses on the children’s necropolis, with 2824 individuals in situ, the vast majority being non-adults (99.0%, 2798/2824), but there were also 26 adults (1.0%). In addition, this necropolis enclosed 15 ossuaries with an estimated minimal number of 57 individuals (37 non-adults and 20 adults). Most inhumations were placed directly in the soil, devoid of organization, and the skeletons, mainly in the supine position, did not obey any specific rule of orientation. Most of the individuals were between 0 and 3 years old (84.6%, 2389/2824), with good to medium preservation, and 47% (1327/2824) presented 75 to 100% of skeletal completeness [38]. Most of the inhumations were single, yet there were some with more than one individual (usually no more than three or four) deposited at a single moment, apparently to make the most use of the space. Furthermore, it was also possible to distinguish graves with more careful inhumations than others [38]. To date, no historical documentation mentions the existence of this children’s necropolis.

These individuals were selected because of the unique characteristics of the site and the availability of well-preserved non-adult skeletons of younger ages, supposedly the age group in which PSL began to occur [32, 39].

Age at death was estimated by standard anthropological methods: the development and dental eruption [40], metric evaluation of the pars basilaris [41], and ossa coxae [42, 43] as well as the diaphyseal lengths of the clavicles [44], humeri [45, 46], and femora [47, 48]. The fusion stages of the skull bones [49], clavicles [50], humeri [50], ossa coxae [49], and femora [51] were also assessed. The individuals were placed in age groups following the suggestions of the US National Institute of Child Health and Human Development [52] and the European Medicines Agency [53] based on several biological and psychological indicators (Table 1).

**Table 1:** Individuals’ age at death distribution by age groups (*N* = 100)

Age at death groups	*n*
Term newborn: birth–28 days	7
Infancy: 1 month–2 years	33
Early childhood: 3–5 years	40
Middle childhood: 6–11 years	20

The biological sex of the individuals was not estimated because it is difficult to observe a correct distinction between sexes in non-adult skeletons [54, 55].

### Elemental Analysis on Bone and Soil with pXRF

Bone elemental analysis followed the guidelines proposed by Gomes et al. [56]. The first step was to identify the regions of the skull (frontal bone, bregma, left and right parietal bones, and the occipital bone), the left and right humeri, and femora (proximal and distal epiphysis, proximal, medial, and distal diaphysis) without lesions or visible taphonomic alternations in which the device’s window was placed. These skeletal regions were chosen given the convex shapes of the skull bones and the thick cortical diaphysis of the humeri and femora that permitted close contact with the pXRF detection window, avoiding air gaps [56]. In every situation, it was assured that the device’s window—with approximately 8 mm of diameter—maintained direct contact with the bone. This was achieved by using the device’s support, or in cases where bones did not fit, foam stabilizers were employed to accommodate bones in the desired position [56]. Additionally, the device’s internal camera allowed the visualization of the window field, ensuring that direct contact was made.

Given that the pXRF’s penetration depth for light elements (Mg to Ca) is close to the surface and that even for heavier elements such as Pb it does not exceed the hundreds of μm [57], the skeletal sites were cleaned superficially with cotton swabs soaked in distilled water and left to dry at room temperature. Additionally, in terms of variations in porosity among different bone regions, Specht et al. [58] indicate that “measurements do not change significantly from bone to bone in cortical bone sites or across the same bone when measured using a portable XRF.” Then, the elemental composition of bone was evaluated using the pXRF model Thermo Scientific Niton XL3t900 with GOLDD + technology. This device has a built-in silver anode functioning at a maximum of 50 kV and 200 μA, with a silicon drift detector that identifies elements from magnesium (Mg) to bismuth (Bi) [59]. The choice of elements to be analyzed in this research was based on significant associations found in previous investigations that measured bone elemental concentrations with pXRF [12, 13, 56]. Thus, the following elements were selected: P, S, Ca, Fe, Sr, and Pb.

A hybrid Compton-fundamental calibration (TestAll) was applied to convert spectral data to quantitative concentrations. According to Zhou et al. [5], there is currently no specific pXRF calibration for organic matrices. In this study, the hybrid Compton-fundamental calibration was selected due to its consideration of the collective amount of unquantifiable light elements with an atomic number below Mg—referred to as balance (%), where oxygen is included [60]. The acquisition time was set at 120 s. For each element, a simple mean (*x̄*) and the standard deviation (*σ*) were calculated, followed by the determination of the coefficient of variation [Cv (%) = (σ/x̄) × 100]. In cases where the Cv was higher than 20% [61], measurements that strongly diverged from the mean were identified and excluded.

Considering the possibility of elemental interchange between soil and bone, the hypothesis of diagenetic contamination was evaluated by analyzing 41 soil samples. These were collected during the excavation by adapting the protocol established for sampling paleoparasitological studies [62]. The soil collected was within the vicinity of the individual’s skull or femora and was then stored in plastic pouches. Thus, each soil sample had a label with the information of the individual and the anatomical region where the sample was collected from.

**Fig. 2:**
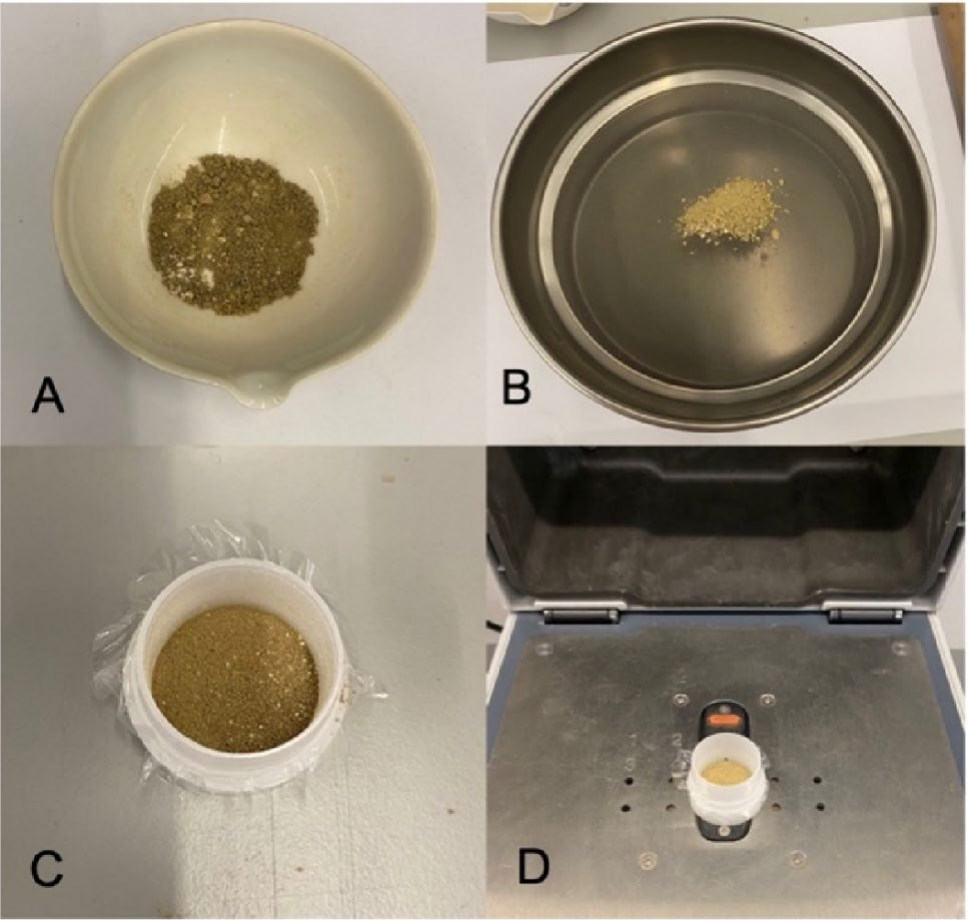
Methodology for sample soil analysis. **A** Soil sample pre-homogenization in the porcelain dish, **B** sample in the 0.5-mm sieve, **C** soil compacted in the cup, **D** sample in analysis by the pXRF

The methodology for the soil preparation was adapted from Williams et al. [60] and performed at the laboratory. The samples were (1) dried for 24 h in an oven at 40 °C, (2) homogenized with a mortar and pestle by hand for approximately 2 min, (3) sieved using a 0.5-mm sieve, (4) placed into sample cups with polypropylene film, and (5) compacted by hand with the help of a small manual rammer tool (Fig. 2). Afterward, using the same experimental framework as the bone samples (TestAll calibration and 120 s of acquisition time), each sample was analyzed in pXRF three times at different points to ensure the correctness of the measure. Then, a single mean was calculated.

### Recording of Porous Skeletal Lesions

The individuals were observed macroscopically with good light conditions, and lesions were recorded as present/absent. An initial observation with the naked eye was later complemented with the help of a magnifying glass (2 × and 4.5 ×), equally important in the decision-making process.

**Fig. 3:**
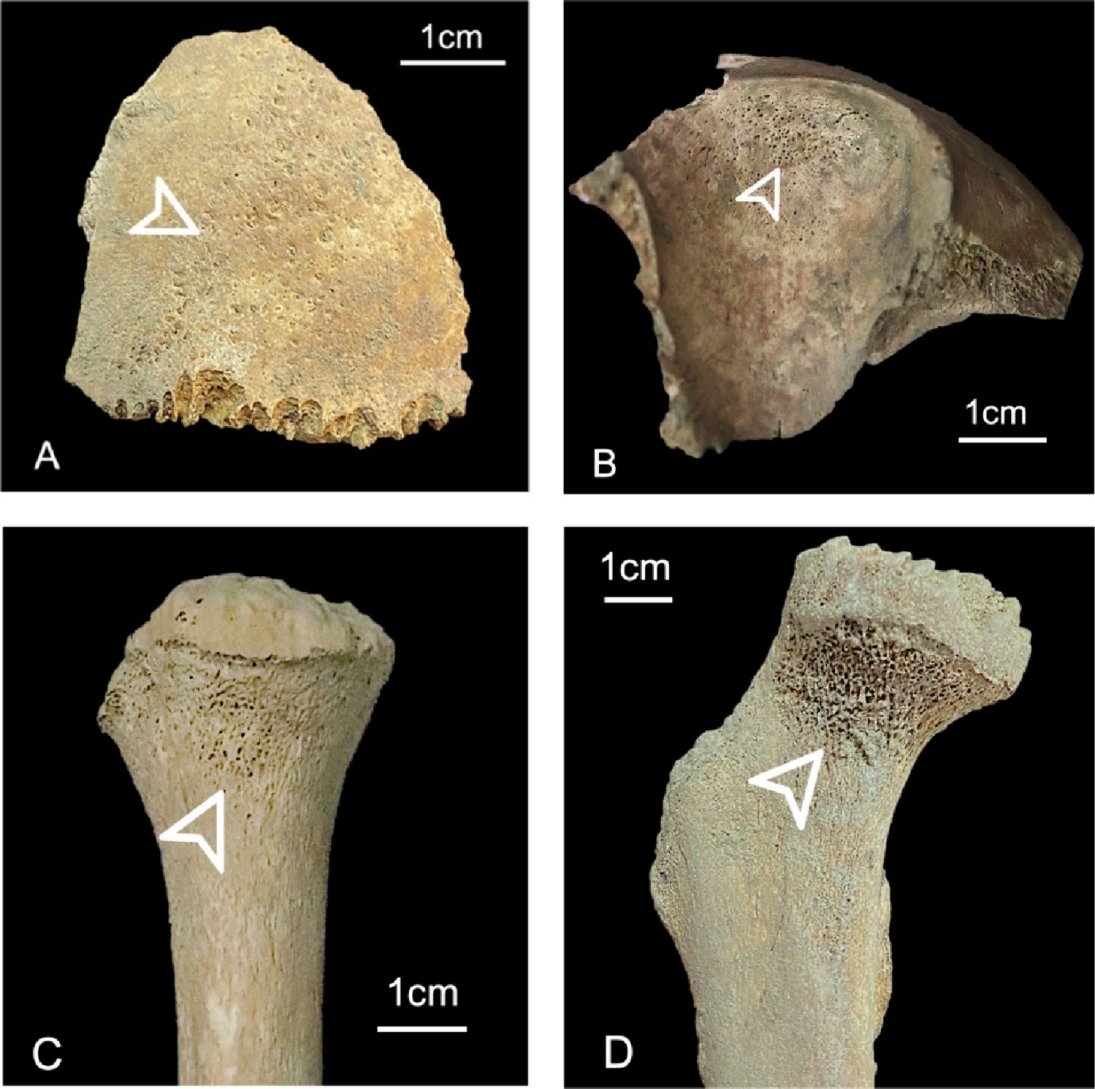
Example of porous skeletal lesions. **A** Cribra cranii on the left parietal of an individual 7–9 years old. **B** Cribra orbitalia in the left orbit of an individual 1–2 years old. **C** Cribra humeralis in the right humerus of an individual 6–8 years old. **D** Cribra femoralis in the right femur of an individual 7–9 years old

Cribra cranii (Fig. 3A) was considered as present when pores were observed in the frontal, parietal, and/or occipital bones whenever at least 50% of the cortical surface was observable. Each bone was divided into four parts to obtain a more precise lesion location [63]. Pores in metabolic active regions, mainly those adjacent to the sutures, and in muscle insertion sites (e.g., occipitofrontalis muscle) were not contemplated as cribra cranii [30].

Cribra orbitalia (Fig. 3B) was recorded if at least one orbital roof, with more than 50% of the surface preserved. Only regions of crescent-shaped and coalescing pores were considered, while pores inserted in acute angles in the posterior margin of the orbit were not registered [64]. Depositions of new bone formation in the orbital were also not classified as cribra orbitalia.

Cribra humeralis (Fig. 3C) and femoralis (Fig. 3D) were recorded as present only when the anatomical neck of the humerus and femur were completely preserved. Thus, only restricted areas of cortical discontinuities located in the medial-anterior region of the neck were considered, excluding porosity in the physeal region [20, 65, 66]. Additional caution was paid to cribra femoralis to avoid confusion with morphological features such as Allen’s fossa or Poirier’s facet [67].

### Testing Data Reliability and Accuracy

A series of steps were taken to guarantee the reliability and accuracy of the collected data, both for the elemental analysis and for cribra recording.

The first step was testing the pXRF device. A standard reference material (SRM)—NIST-1400, constituting of homogenized human bone ash with certified elemental concentrations—was measured under identical experimental circumstances to the bone samples [56, 68]. Then, the relative technical error of measurement (rTEM) was calculated:

$${\text{rTEM}}(\mathrm{\%})=({\text{TEM}}/{\text{mean}})\mathrm{ x }100$$

Thirty measurements were obtained in two different moments from ten randomly selected individuals. To ensure proper intra-observer replicability, the rTEM should be equal to or lower than 10% [69]. Additionally, the coefficient of reliability (CR) was computed.

$${\text{CR}} \, \text{=}\frac{{\text{SD}}^{2}}{{\text{SD}}^{2}+ \text{ } {\text{TEM}}^{2}}$$

When interpreting the CR, values closer to 1 or 0 indicate higher or lower reliability or consistency of measurements, respectively.

The second step was the evaluation of possible contamination and elemental interchanges with soil. The concentrations of the elements under study were compared for soil and bone. Forty-one individuals were selected (corresponding to the same individuals from where the soil was collected), and three measurements were made for each individual—in the skull, humerus, and femur—then, a single mean was computed.

The third step was the estimation of intra- and inter-observer errors: As recommended during the International Meeting on Porous Skeletal Lesions [70], 20 individuals were randomly selected to calculate these errors. The first and second evaluations of intra-observer error were made 18 months apart.

The previous steps were part of a pre-analytical phase of the research to ensure that the concentrations of the elements under analysis resulted from the individual’s composition and were not significantly influenced by the type of soil. At the same time, it was attested that the recording of the four cribra was carried out consistently.

### Analytical Phase

The analytical phase was focused on evaluating potential variations in bone elemental concentration based on the presence/absence of cribra. To achieve this, the researchers tested whether there were differences in the concentration of elements between individuals with and without cribra. If so, they evaluated whether these differences depend on the type of cribra by comparing the elemental concentration levels in individuals with and without a specific type of cribra.

### Statistical Analysis

The intra- and inter-observer errors were evaluated using Cohen’s kappa value [71]. According to McHugh [72] kappa values should be interpreted as (1) without agreement, between 0 and 0.20; (2) minimal, 0.21 to 0.30; (3) weak, 0.40 to 0.59; (4) moderate, 0.60 to 0.79; (5) strong, 0.80 to 0.90; and (6) almost perfect, above 0.90. Any kappa below 0.60 indicates inadequate agreement.

In cases where the mean of elemental concentration was calculated (e.g., presence of PSL), the standard error of the mean (SEM) was preferred to the standard deviation as these means were themselves calculated from aggregate data. The SEM provides a measure of precision that accounts for the data’s sampling variability [73].

Attending to the sample size (*N* = 100), the normality of the distribution of continuous variables, such as the elemental concentration, was assessed via the Kolmogorov–Smirnov test. Possible associations between elements of bone and soil as well as variations in elemental concentration with the presence of PSL were evaluated using the parametric Student’s *t*-test (normal distribution) or its non-parametric alternative, the Mann–Whitney *U*-test (non-normal distribution). The one-way ANOVA (normal distribution) and the Kruskal–Wallis test (non-normal distribution) were used to test for age-at-death associations [74]. The possible association between the PSL and the groups of age at death was measured using the chi-square test (χ2).

Furthermore, a multivariate approach was applied. Initially, principal component analysis (PCA) was used to identify which elements were more closely related (components). The internal consistency was guaranteed by the “eigenvalue higher than 1” rule, and a varimax rotation was performed [74]. Nevertheless, considering that elemental data is considered to be closed [75], concentrations were previously centered log-ratio transformed, allowing the observation of clear patterns in the PCA [76].

Then, a binary logistic regression was computed. This test presents the odds ratios (ORs) for multiple explanatory variables, measuring associations and risk factors for a specific outcome [77, 78]. In this case, the researchers evaluated which groups of elements (PCA components) better predict the expression of PSL, controlling for age at death. An OR equal to 1 indicates no effect, an OR greater than 1 suggests higher odds, and an OR less than 1 signifies lower odds of the outcome. A 95% confidence interval (CI) estimates OR precision; wider CIs indicate lower precision. Significance was set at *p* < 0.05. The analysis was performed in IBM SPSS Statistics® 29.0 (IBM Corp., Armonk, NY, USA).

## Results

### Pre-analytical Phase: Testing the Adopted Procedures

The accuracy of the pXRF device was confirmed by contrasting the certified values of the SRM-1400 with those measured by the pXRF (Table S1). Additionally, Table 2 shows that the rTEM between the two measurements was below 5% for all the elements. For each element, the associated reliability coefficients were close to 1. Sulfur and Ca presented the best results, while Si and Fe performed less well.

**Table 2:** Results of the relative technical error of measurement (rTEM) and the associated reliability coefficients calculated for the elements under analysis

Elements	rTEM (%)	Reliability coefficient
Si	4.76	0.99
S	1.48	0.98
P	3.70	0.99
Ca	1.47	0.97
Fe	4.51	0.99
Sr	2.69	0.97
Pb	3.78	0.99

Furthermore, possible elemental interchanges between soil and bone were evaluated to exclude contamination. As illustrated in Table 3, the concentration of all the elements is significantly different between soil and bone. Phosphorus, S, Ca, and Sr have higher concentrations in bone, while Fe and Pb present higher concentrations in soil. The ratio Ca/P was also higher in bone.

**Table 3:** Comparison of the mean elemental concentration (± SEM) for the six elements under analysis and the Ca/P ratio in bone (*n* = 41) and soil (*n* = 41). Values are in g/kg except for Sr and Pb (mg/kg)

	**P**	S	**Ca**	**Fe**	**Sr**	**Pb**	**Ca/P**
Bone	148.8 ± 1.6	3.9 ± 0.2	315.3 ± 2.7	1.4 ± 0.2	293.2 ± 10.6	188.1 ± 12.8	2.12 ± 0.01
Soil	5.7 ± 0.3	0.7 ± 0.1	95.7 ± 3.7	18.3 ± 0.3	101.9 ± 2.42	284.3 ± 27.6	18.1 ± 1.01
Sig.	** < 0.001**	** < 0.001**	** < 0.001**	** < 0.001**	** < 0.001**	**0.014**	** < 0.001**

Statistically significant results are in bold (significance set at *p* < 0.05)

Altogether, these results seem to indicate that the elemental data obtained can be trusted and used in further analysis.

Concerning the replicability of the recording of cribra’s presence/absence, the estimation of the intra- and inter-observer errors indicates nearly perfect agreement for cribra orbitalia and strong to moderate agreement for cribra cranii, humeralis, and femoralis (Table 4). In each instance, the concordance surpassed 0.60, validating the cribra recording.

**Table 4:** Results of the intra- and inter-observer error assessed by Cohen’s *K* in 20 individuals for the four cribra

**PSL**	**Intra-observer error**		**Inter-observer error**	
	Cohen’s *K*	Sig.	Cohen’s *K*	Sig.
Cribra cranii	0.78	** < 0.001**	0.69	**0.001**
Cribra orbitalia	0.91	** < 0.001**	0.91	** < 0.001**
Cribra humeralis	0.83	** < 0.001**	0.86	** < 0.001**
Cribra femoralis	0.78	** < 0.001**	0.89	** < 0.001**

Statistically significant results are in bold (significance set at *p* < 0.05)

### Analytical Phase

Out of the 100 individuals analyzed, in four, it was impossible to ensure whether the visible changes could be registered as cribra. Thus, the motto “In case of doubt, do not consider” was adopted. However, since these individuals had soil samples, they were kept in the sample. From the remaining 96 individuals, 69.8% (67/96) presented at least one type of cribra, and 30.2% (29/96) were registered as without cribra (Figure S1). As seen in Table 5, individuals with cribra presented significantly higher concentrations of P and Ca and lower concentrations of Fe than those without cribra.

**Table 5:** Mean elemental concentration (± SEM) values of individuals with (*n* = 67) and without (*n* = 29) cribra, with cribra cranii (*n* = 19), cribra orbitalia (*n* = 42), cribra humeralis (*n* = 13), and cribra femoralis (*n* = 46). Values are in g/kg except for Sr and Pb (mg/kg)

	**P**	**S**	**Ca**	Fe**Fe**	**Sr**	**Pb**	**Ca/P**
**With cribra**	150.5 ± 1.1	4.1 ± 0.2	319.5 ± 2.2	1.2 ± 0.1	289.2 ± 7.36	192.0 ± 9.29	2.13 ± 0.01
**Without cribra**	142.2 ± 2.6	3.7 ± 0.1	307.9 ± 4.7	2.0 ± 0.4	295.2 ± 13.8	191.3 ± 15.4	2.17 ± 0.02
Sig.	**0.006**	0.09	**0.01**	**0.04**	0.98	0.97	0.06
**Cribra cranii**							
Present	149.7 ± 2.3	4.2 ± 0.3	317.2 ± 4.2	1.4 ± 0.3	285.0 ± 14.8	182.9 ± 14.1	2.12 ± 0.02
Absent	148.2 ± 1.4	4.0 ± 0.1	316.3 ± 2.6	1.3 ± 0.1	290.0 ± 8.1	194.2 ± 10.4	2.14 ± 0.01
Sig.	0.62	0.52	0.87	0.70	0.77	0.53	0.52
**Cribra orbitalia**							
Present	149.9 ± 1.5	4.2 ± 0.2	319.0 ± 2.7	1.2 ± 0.1	278.0 ± 7.5	192.9 ± 12.5	2.13 ± 0.01
Absent	145.5 ± 2.4	3.8 ± 0.2	312.6 ± 4.4	1.5 ± 0.2	303.7 ± 14.4	182.6 ± 11.6	2.15 ± 0.02
Sig.	0.10	0.11	0.20	0.15	0.12	0.56	0.25
**Cribra humeralis**							
Present	151.4 ± 2.3	3.9 ± 0.4	323.6 ± 5.1	1.5 ± 0.4	316.1 ± 18.8	174.4 ± 20.5	2.14 ± 0.13
Absent	146.4 ± 1.5	3.9 ± 0.1	314.2 ± 2.8	1.6 ± 0.2	289.7 ± 8.3	190.9 ± 10.5	2.15 ± 0.01
Sig.	0.16	0.89	0.16	0.72	0.20	0.51	0.67
**Cribra femoralis**							
Present	151.5 ± 1.3	4.1 ± 0.2	320.8 ± 2.5	1.2 ± 0.1	296.5 ± 9.3	174.3 ± 9.7	2.12 ± 0.01
Absent	142.8 ± 1.8	3.9 ± 0.1	310.9 ± 3.7	1.9 ± 0.3	292.6 ± 10.4	212.6 ± 15.1	2.17 ± 0.01
Sig.	** ≤ 0.001**	0.47	**0.03**	**0.02**	0.78	**0.03**	**0.001**

Statistically significant results are in bold (significance set at *p* < 0.05)

The concentration of the elements was also assessed for individuals with specific types of cribra and compared to those that did not present these cribra, i.e., with distinct cribra co-occurrence. In this case, the most frequently observed lesion was cribra femoralis (47.9%, 46/96), followed by cribra orbitalia (43.8%, 42/96), cribra cranii (19.8%, 19/96), and cribra humeralis (13.5%, 13/96). Statistically significant differences were only found for individuals with cribra femoralis, presenting higher levels of P and Ca and a lower Fe, Pb, and Ca/P ratio (Table 5). Even if the results were only statistically significant for cribra femoralis, similar trends occur for cribra orbitalia and cribra humeralis. In contrast, individuals with cribra cranii present higher levels of Fe.

The elemental data and the Ca/P ratio (as a passive variable) were further analyzed using a multivariate approach. A PCA identified three components explaining 80.9% of the total variance (Table S2). Principal component 1 (Pc1) was characterized by a strong positive association with P, Ca, and Sr and a negative association with the Ca/P ratio and explained 48.5% of the variance. Principal component 2 (Pc2) was positively associated with S and negatively associated with Fe, representing 18.4% of the total variance. Lastly, principal component 3 (Pc3) presented a strong association with Pb, accounting for 13.9% of the total variance.

The results of the one-way ANOVA show that both Pc1 (*p* = 0.002) and Pc2 (*p* = 0.004) had a significant association with the groups of age at death (Table 6). In this case, for statistical purposes, the “term newborn” group (birth–28 days) with seven individuals was merged with the “infancy” group (1 month–2 years). It was observed that individuals aged 2 years or younger (infancy) presented significantly lower concentrations of P, Ca, and Sr (Pc1). Contrariwise, in early (3–5 years) and middle childhood (6–11 years), Fe levels decreased, while S levels increased (Pc2).

**Table 6:** Test on the scores of the principal components (Pc1, Pc2, Pc3) according to groups of age at death and presence of cribra cranii (*n* = 19), cribra orbitalia (*n* = 42), cribra humeralis (*n* = 13), and cribra femoralis (*n* = 46)

	**Pc1 (P, Ca, Sr)**	**Pc2 (S, Fe)**	**Pc3 (Pb)**
Groups of age at death	***p* = 0.002** Early and middle childhood > P, Ca, Sr	***p* = 0.004** Early and middle childhood < Fe; > S	*p* = 0.10
Cribra cranii	*p* = 0.48	*p* = 0.69	*p* = 0.79
Cribra orbitalia	*p* = 0.82	***p* = 0.03** With CO < Fe; > S	*p* = 0.38
Cribra humeralis	*p* = 0.15	*p* = 0.94	*p* = 0.78
Cribra femoralis	***p* < 0.001** With CF > without CF	***p* = 0.04** With CF < Fe; > S	*p* = 0.70

Statistically significant results are in bold (*p* < 0.05)

Moreover, a significant association was found between Pc1 and the presence of cribra femoralis (*p* < 0.001), meaning that individuals with this lesion have higher levels of P, Ca, and Sr (Table 6). Pc2 was also significantly associated with the presence of cribra orbitalia (*p* = 0.03) and cribra femoralis (*p* = 0.04); here, individuals with these lesions had lower concentrations of Fe and higher concentrations of S (Table 6).

A logistic regression was then applied, where the principal components extracted from the PCA and the groups of age at death were the predictive variables for the expression of cribra (Table S3). The results show that except for cribra cranii, the age at death increases the odds of expressing cribra orbitalia (OR = 1.86; CI = 0.94–3.68), cribra humeralis (OR = 8.32; CI = 2.71–25.60), and cribra femoralis (OR = 6.97; CI = 2.78–17.45). Similar results were already observed in the univariate statistical analysis (Supplementary Text 2, Table S4). Additionally, higher levels of P, Ca, and Sr (Pc1) increased the odds of having cribra femoralis (OR = 2.30; CI = 1.23–4.29). Lastly, the odds of exhibiting cribra orbitalia (OR = 1.76; CI = 0.97–3.20) and cribra femoralis (OR = 1.42; CI = 0.73–2.74) significantly increased in individuals with a lower concentration of Fe and a higher concentration of S (Pc2).

## Discussion

The main aim of this investigation was to assess whether bone elemental concentration contributes to the interpretation and discussion of the etiology(ies) of PSLs. The analyses carried out by pXRF, showing the measured concentrations obtained in the SRM-1400, are similar to those certified by the National Institute of Standards and Technology (NIST), which increases the reliability of the elemental data collected. Moreover, the results of the rTEM lower than 5% and the CR closer to 1 allow to state that the elemental data is trustworthy.

One of the main issues when analyzing elemental concentrations from archaeological bones is the possibility of elemental interchanges with the soil [79], which could contaminate the bone under analysis. Even if some studies have applied pXRF to study the elemental composition of archaeological soils (e.g., 80) to the authors’ knowledge, this research is pioneering in applying this technique to compare elemental concentrations of the soil found near the individual’s skull and femora. The soil samples were measured under the same experimental protocol as the bones, and all the elements under study presented statistically significant different concentrations between soil and bone (Table 3). This reduces the possibility of contamination that could otherwise impact the accuracy of the results.

As expected, P, S, Ca, and Sr presented higher concentrations in the bone. Calcium and P are the main constituents of bone mineral content [81], and their levels in the sample (Ca = 315.3 ± 2.7 g/kg; P = 148.8 ± 1.6 g/kg) are similar to those observed by Gomes et al. [14] in non-adult individuals from identified collections (Ca = 257.8 ± 1.3 g/kg; P = 130.4 ± 1.1 g/kg) and by Zaichick and Zaichick [82] in a fresh bone from a biopsy (Ca = 218 ± 10 g/kg; P = 112 ± 9 g/kg). The ratio Ca/P, as reported in other studies, gives an insight into bone preservation since it measures the integrity of hydroxyapatite [83, 84]. Usually, in bone samples, Ca/P ranges from 1.94 to 2.92 (see Table 4 in 12), which coincides with the value obtained here (Table 3, 2.12 ± 0.01).

Sulfur, after Ca and P, is the most abundant mineral element in the human body [85]. In this research, S levels in bone (3.9 ± 0.2 g/kg) were analogous to those in fresh non-adult bones [~ 2.5 g/kg; 86]. Lastly, Sr not only tends to accumulate in the bone [87] but also has a great affinity with Ca, with the potential to replace it during the growth period [88]. In this work, Sr levels (293.2 ± 10.6 mg/kg) were within the range (~ 43 to 381 mg/kg) of those found in other studies with non-adult skeletal samples [2, 14, 83, 89–91]. These results suggest the minor effect of diagenesis in the studied sample.

On the other hand, Fe and Pb levels were higher in the soil. Iron is known to be present in most soils [92, 93], and its presence, particularly in bones from archaeological individuals, may be conditioned by diagenetic effects. The similarity of Fe levels obtained here (1.4 ± 0.2 g/kg) with the values reported by Martínez-García et al. [94] in non-adult autopsied bones (0.5 to 1.5 g/kg) and by Gomes et al. [14] in non-adult identified skeletons (1.7 ± 0.3 g/kg) suggests that the Fe present in the soil (18.3 ± 0.3 g/kg) did not contaminate bone significantly. Additionally, Fe is present in bone tissue without disturbing the apatite structure and bone function [95]. Studies with radionuclides showed that among others Fe is a bone-seeking element [96]. As bone turnover is slow, the biological half-lives of the elements within it are estimated to be up to 10 years [97]. Thus, bone is a suitable indicator for the evaluation of low-level and long-term intake and deposition of Fe absorbed through diet.

The case of Pb is slightly different. First, in Lisbon’s area, where the samples are from, the soil has high levels of Pb, reaching concentrations of up to 585 mg/kg [98]. Indeed, the Pb concentration in this sample was much higher (188.1 ± 12.8 mg/kg) than that reported in fresh non-adult bones (11.0 ± 32.7 mg/kg; [99]) and in identified skeletons (28.3 ± 2.4 mg/kg; [14]). Moreover, the PCA results show Pb separately represented by Pc3 with total lack of association with other elements (Table S2). Altogether, this seems to point to a diagenetic effect of Pb in bones, meaning that this element should be discarded from further analysis.

After assuring that the elemental data obtained by pXRF was reliable and could be used in the subsequent analysis, the next step was to evaluate whether the recording of cribra was consistent. The value of Cohen’s *K* for the intra- and inter-observer errors (Table 4) was always above 0.60 (the minimum value was 0.69 obtained for cribra cranii in the inter-observer error), meaning that the four cribras were correctly registered.

The results of the pre-analytic phase show that except for Pb, data from both the elemental concentrations and the cribra recording was reliable, which allowed to move to the analytical phase.

Individuals with cribra (68.8%, 67/96) presented statistically significantly higher concentrations of P and Ca and lower concentrations of Fe than those without cribra (30.2%, 29/96). Similar results were found for cribra femoralis (Table 5). While this cribra was the only one with statistically significantly higher values of P and Ca and lower concentrations of Fe, a closer look at the data allows to observe that cribra orbitalia and humeralis follow a similar trend. Miquel-Feucht et al. [66] previously suggested the same etiology for these three cribra based on similar macroscopic, radiologic, and histological features, leading these authors to propose a cribrous syndrome. The elemental data observed in this research seems to point in the same direction. Nevertheless, it is not possible to reach a definitive conclusion since most macroscopic studies, with non-adult samples, have not yet fully proven the existence of such a syndrome [34, 65, 100].

The elemental variations found in individuals with cribra may be the result of an association with the age of the individuals. In this sample, the ages at death were much younger than those analyzed in other studies. For example, the identified individuals studied by Gomes et al. [14] had a mean age at death of 13.2 years old (from 0 to 20 years), while in this research, 41.7% (40/96) of the individuals were aged 5 years or younger, and the eldest was 11 years old. Moreover, most of the individuals without lesions (72.4%, 21/29) were less than 2 years old, while cribra orbitalia and femoralis were recorded in individuals older than 1 to 2 years and cribra cranii and humeralis in individuals older than 3 years. These results align with the biological approach proposed by Brickley [31, 39] for the expression of PSL. If marrow hyperplasia is behind cribra’s expression, these lesions should be more common in younger ages—particularly under 11 years old, the age when the skull, humeri, and femora enclose red active marrow that is actively producing RBC [101]. Overstimulated RBC production may occur in situations of higher demand, such as in anemic states, where oxygen flow is insufficient, potentially leading to marrow hyperplasia [31, 39]. Likewise, in this research, cribra was not registered before the first year of life, which coincides with the lesion’s window formation proposed by O’Donnell et al. [32]. The elemental concentrations of P and Ca show a positive association with age, while Fe decreases in the early and middle childhood age groups (older than 2 years).

The multivariate approach corroborates these results. Pc1 (Ca, P, and Sr) was significantly associated with cribra femoralis, and in the logistic regression, individuals with higher concentrations of Ca, P, and Sr (Pc1) presented higher odds of expressing this cribra (OR = 2.30; CI = 1.23–4.29). Thus, the elemental variations may be more associated with an age effect than with the expression of cribra itself. Evidence of this comes from the fact that a positive age trend was already reported for the concentrations of P, Ca, and Sr in identified non-adult skeletons [14], while in adults, the concentration of these elements decreases with age [12]. Physiologically, P and Ca concentrations exhibit an upward trend during growth [102, 103]. Generally, they begin to decline in middle to late childhood, only to rise again in adolescence [104]. Strontium, given its affinity with Ca [87], follows a similar trend, peaking in the growth spurt to then decline, stabilizing in the late 20s [105]. Eighty-two percent (77/96) of the individuals from the present sample were aged 6 years or less, which fits the profile for the age-related changes of Ca, P, and Sr in the human body.

However, why was this elemental variation only observed in the individuals with cribra femoralis? While age at death was found to significantly increase the odds of expressing cribra orbitalia (OR = 1.86; CI = 0.94–3.68) and cribra humeralis (OR = 8.32; CI = 2.71–25.60), the age increment of cribra femoralis is particularly distinctive (Table S3). In infancy, 18.9% (7/37) of individuals exhibit this cribra, a percentage that rises to 57.5% (23/40) in early childhood, peaking in middle childhood (84.2%, 16/19). This contrasts with cribra orbitalia, with similar frequencies in early (50.0%, 20/40) and middle (52.6%, 10/19) childhood. Also, cribra humeralis was not recorded in individuals aged less than 3 years, and in middle childhood, only 36.8% (7/19) of individuals present this lesion. This continuous and increasing presence of cribra femoralis across the different age groups may justify why the statistical tests only detect significant differences in the concentrations of Ca, P, and Sr for this cribra. Further research is needed to better understand the association of cribra with higher levels of these elements in non-adult bones.

The multivariate approach results also showed that Pc2—higher concentrations of Fe and lower concentrations of S—increases the odds of expressing cribra orbitalia (OR = 1.76; CI = 0.97–3.20) and cribra femoralis (OR = 1.42; CI = 0.73–2.74). While Pc2 exhibited an association with the age at death—older individuals presented higher levels of S and lower levels of Fe—it seems more of an indirect connection, in this case more associated with the expression of cribra orbitalia and cribra femoralis.

The association of Fe and S with cribra was already observed in identified non-adults [14] and adults [12]. Nevertheless, in both studies, higher concentrations of Fe and lower concentrations of S were seen in association with cribra cranii. In this research, the concentration of Fe, while not statistically significant, was also higher in individuals with cribra cranii, contradicting the tendency observed for the other three cribra. This is where age could be a relevant factor since both studies analyzed individuals older (respectively, *x̄* = 13.2 years; *x̄* = 47.1 years) than those in this research. It has been suggested that cribra cranii requires more time to progress in the face of homeostatic ruptures [32]. Flat bones of the cranial vault take more time to develop changes and remodel [106, 107], which can extend the expression period of cribra cranii. Moreover, the skull preserves red bone marrow, especially in the parietals, where cribra cranii is more frequent, later than the postcranial skeleton, usually until 11 to 15 years [39, 108, 109]. A need for more RBC may lead to marrow expansion, which, in combination with abnormal bone remodeling, may result in cribra expression [110].

Iron concentration being higher for individuals with cribra cranii and lower for individuals with cribra orbitalia and cribra femoralis may also be related to the age expression of the lesions. Gomes et al. [12, 14] suggested that when a diet is lower in Fe and storage levels in the body decrease, the production of hepcidin (the hormone responsible for controlling Fe absorption) lowers, and the absorption of Fe increases up to 40% [111, 112]. It is possible that the compensatory mechanism of Fe accumulation only takes place after some time, explaining why there are low Fe levels in individuals with cribra orbitalia and femoralis (these lesions expressed earlier) and higher in individuals with cribra cranii (these lesions expressed later).

Other investigations have also found an association between low Fe and cribra orbitalia in non-adult and adult individuals [e.g., 2, 10, 111, 113, 114], arguing in favor of an iron deficiency anemia (IDA) origin for the lesion. Nevertheless, the current state of the art on PSL is moving away from a straightforward connection between IDA and cribra [27, 31, 115, 116], and other options must be pointed out.

Anemia of chronic disease (ACD) or anemia of inflammation refers to impaired RBC production associated with chronic or acute and severe inflammatory states [117]. The inflammatory response, particularly in severe bacterial infections, enhances the production of hepcidin, drastically lowering Fe levels [118]. Nevertheless, the active macrophages, owing to the inflammatory condition, consume senescent erythrocytes, degrading hemoglobin, and storing the liberated Fe, leading to a possible Fe overload [119]. The authors further state that ferritin levels can either be maintained or higher in ACD, meaning that depending on the infection phase, lower and higher levels of Fe can be found. O’Donnell et al. [32], based on the analysis of CT scans of non-adult crania, observed that an initially inflammatory response associated with more severe but acute conditions can result in cranial porosity, while marrow expansion would be verified in more chronic systemic disorders. Cribra orbitalia was seen to not result only from marrow expansion, while cribra cranii was more common when hyperplasia was present [110].

Bone marrow in ACD can be hypocellular, normocellular, or hypercellular [120]. There is often erythroid hypoplasia, although the erythropoiesis can be normal or even increased, and the myeloid and megakaryocytic are frequently increased, leading to hyperplasia [121]. Additionally, ACD seems to fit with previous findings of higher odds of expressing cribra in non-adult individuals with respiratory infections, such as pneumonia and pulmonary tuberculosis, both with acute and chronic phases [32–34]. Herein, it could be possible, depending on the state of inflammation/infection and the age of onset for the lesion formation, to find associations among cribra cranii, high Fe levels, and more chronic conditions as well as among cribra orbitalia and femoralis, low Fe, and acute but severe inflammations.

Another condition is sideroblastic anemia (SA) characterized by abnormal erythroid precursors [122], particularly, acquired SA results of nutritional impairments such as deficiencies in vitamin B6, B9, and B12, the ingestion/accumulation of toxins, and alcohol abuse [123, 124]. In fact, SA was associated with cribra cranii and high Fe concentration in non-adult identified skeletons [14]. This type of anemia not only was clinically correlated with Fe overload but also echoes poor nutrition and early alcohol use [12, 14]. This aligns with Portugal’s 19- to 20th-century living conditions, where children often sucked rags soaked in milk and wine and were fed bread mixed with honey, water, and wine [125].

It cannot be overlooked that the association between cribra orbitalia and femoralis and low Fe does not necessarily mean causation. Several factors can be responsible for the expression of PSL [2]. There is always the possibility of comorbidity, and several conditions may be affecting an individual at the same time. For instance, low Fe content may originate from the low consumption of iron-rich foods that are usually correlated with other nutritional deficiencies, such as those of B-complex vitamins.

Thus, the possibility of megaloblastic anemias (MAs) should be explored. These conditions are characterized by the presence of large RBC precursors called megaloblasts in hyperplasic bone marrow [126]. The most common causes are deficiencies of vitamin B9 and vitamin B12 [127], not only in cases of the lack of ingestion of animal-source foods, but also in cases of the malabsorption of these vitamins because of gastric complications [128], as in parasitic infections [129]. Individuals with MA may express mildly elevated concentrations of total Fe, yet it is not unusual to observe normal Fe stores because bone marrow is overloaded with Fe that cannot be used during the megaloblastic state [130]. Moreover, it is common to find MA in co-occurrence with IDA [131]. In some cases, IDA may even precede the development of MA, and low Fe levels are a concomitant finding in MA [132, 133]. Walker et al. [27] had already proposed that MA may be behind cribra’s expression, within a generalized framework of nutritional deficiencies, including the lack of vitamin C, which decreases non-heme Fe fixation from vegetable food sources [134].

Following this reasoning and considering the studied non-adult sample, it is important to mention that prior to weaning, most of the necessary Fe and vitamins are received from maternal milk. A mother with nutritional deficiencies will not be able to supply adequate nutrition to her children, possibly leading to IDA, MA, or even other forms of anemia and pathologies [27]. Moreover, both human’s and cow’s milk have low concentrations of Fe, yet the availability of this element is greater in human milk [135]. Children fed with cow’s milk present an increasing risk up to 39% to develop IDA [136]. Infants can also experience microscopic blood loss given the proteins in cow’s milk that induce colitis. This chronic blood loss contributes to severe Fe deficiency and can impair the absorption of vitamin B9 and B12, leading to MA [128].

In 19th-century Portugal, life conditions were harsh for the general population but particularly unkind for infants and children, with a significant number of foundlings [137]. Historical sources report that breastfeeding was less than ideal as mothers were often malnourished and ill [138]. Foundlings were frequently fed by wet nurses, yet these women were paid quite less than those working for the upper classes, and in many cases, they rationed the milk to accommodate multiple children [139]. This had health implications as both wet nurses and children suffered from infections, which spread easily [140]. Thereby, even if the age of weaning was set around 2 years old, it often happened much earlier in the lower social strata [141]. The inclusion of animal milk—especially cow’s and goat’s milk—was quite common, and some authors refer to it as an omnipresent food in the diet of infants and children [142]. According to Lopes [141], this led to clinically reported gastrointestinal issues associated with high mortality. This socio-economical profile and lifestyle seem to agree with the physiological framework presented earlier, with complex interactions mainly caused by nutritional deficiencies.

Adding to the discussion is also the fact that individuals with cribra orbitalia and cribra femoralis presented higher concentrations of S. This element is mostly integrated through food [143], and low sulfur amino acid values were observed in individuals’ low consumption of red meat and animal protein [85], which appears not to be the case here. Briefly, two possibilities are presented to explain higher sulfur in these individuals. First, S is present in certain amino acids, such as cysteine and methionine [144]. Cysteine, in particular, plays a crucial role in forming disulfide bonds and stabilizing the structure of proteins and is involved in the synthesis of ferritin and transferrin [145]. Thus, in the case of a higher demand of these proteins, such as in anemia, higher cystine production can raise generalized sulfur content [146]. Additionally, it was observed that the deficiency of folate (vitamin B12) leading to MA is associated with high levels of homocysteine [147]. Second, although less likely, it is possible that the individuals under analysis were receiving adequate nutritional intake, with a proper content of Fe, S, and vitamins. However, gastric conditions could prevent the absorption of Fe and vitamins but not of S, which could explain in the univariate approach, no differences were found in the S concentration between individuals with or without cribra. Either way, both hypotheses point to individuals with severe homeostatic ruptures.

## Conclusions

This study showed the usefulness of pXRF for studying the elemental concentration of human bones. The Thermo Scientific Niton XL3t900 model correctly determined the concentrations of the SRM with low technical errors of measurement.

Previous studies have raised the possibility of bone tissue contamination with elements from the soil. The pioneering procedures used in this work—both the elemental analysis of soil in contact with the individuals’ bones and the cleaning of the bone surface with distilled water—proved the reliability of elemental concentrations obtained in these individuals. This also allowed to exclude elements that contaminate bone, such as Pb. Furthermore, the Ca/P ratio confirmed the preservation of bone integrity.

The elemental variations observed in this sample of non-adults indicate that individuals with cribra have lower concentrations of Fe and higher concentrations of S when compared with individuals without cribra. This pattern was also demonstrated with statistical significance in individuals with cribra femoralis (47.9%, 46/96). A similar trend was observed among individuals with cribra orbitalia and cribra humeralis, although without statistical significance. These findings support the hypothesis of a shared etiology among these three types of cribra, as suggested by other researchers. Additionally, they may indicate a distinct origin for cribra cranii given that individuals with this cribra exhibited higher Fe concentrations.

The multivariate approach corroborates these results as the odds of exhibiting cribra orbitalia (OR = 1.76; CI = 0.97–3.20) and cribra femoralis (OR = 1.42; CI = 0.73–2.74) significantly increased in individuals with lower concentrations of Fe and higher concentrations of S. This result contradicts the findings obtained in previous studies. While low Fe levels have been linked with cribra and IDA, the current understanding of PSL suggests more complex associations. Factors such as ACD as well as SA and MA may contribute to the development of these lesions. This highlights the multifaceted nature of their etiology and the importance of considering broader nutritional and health contexts in their interpretation.

Another important finding is that higher levels of P, Ca, and Sr were associated with increased odds of individuals developing cribra femoralis (OR = 2.30; CI = 1.23–4.29). Age significantly influences the concentrations of P, Ca, and Sr, with older individuals demonstrating higher levels of these elements, which aligns with the physiological processes of skeletal growth during the developmental stages. The results support the hypothesis that the age at death increases the frequency of cribra as the odds of individuals exhibiting cribra orbitalia (OR = 1.86; CI = 0.94–3.68), cribra femoralis (OR = 6.97; CI = 2.78–17.45), and cribra humeralis (OR = 8.32; CI = 2.71–25.60) increased with age.

Utterly, these results significantly broaden cribra research and interpretation, deepening the understanding of PSL, with the potential to offer valuable guidance in clinical research.

## Supplementary Information

Below is the link to the electronic supplementary material.

Supplementary file1 (DOCX 138 KB)

## Data Availability

No datasets were generated or analyzed during the current study.
