# Supplementary material for: The Role of Fe, S, P, Ca, and Sr in Porous Skeletal Lesions: A Study on Non-adult Individuals Using pXRF
Source: Biol Trace Elem Res. 2024 May 1;203(2):591–607. doi: 10.1007/s12011-024-04187-4 (PMC11750918; doi:10.1007/s12011-024-04187-4)
Supplement: Supplementary file 1 — Supplementary file1 (DOCX 138 KB) [file 12011_2024_4187_MOESM1_ESM.docx]

**Supplementary information**

|  | **Elemental concentration** | | | | | | | |
| --- | --- | --- | --- | --- | --- | --- | --- | --- |
|  | **Si** | **P** | **K** | **Ca** | **Fe** | **Zn** | **Sr** | **Pb** |
| **pXRF**  **S.D.** | 0.12  (0.02) | 18.80  (0.22) | 195.6 (48.8) | 38.09  (0.47) | 555.57  (35.88) | 179.50 (5.72) | 242.40  (0.3) | 10.57  (2.13) |
| **Certified Value**  **S.D.** | 0.13 | 17.91 (0.19) | 186.0  (8.0) | 38.18 (0.13) | 660.0  (27.0) | 181.0  (3.0) | 249.0  (7.0) | 9.07  (0.12) |

**Table S1.** Comparison of the mean elemental concentration obtained with the pXRF and the certified concentration (standard deviation – S.D.) of the bone ash SRM NIST-1400 (measured 15 times). Concentrations are in µg/g except for Si, P, and Ca (wt%).

**Supplementary text 1**

The co-occurrence of different types of cribra was evaluated (Figure S2). Thirty individuals (31.3%) presented only one lesion, while 37 presented more than one (38.6%). The most common combination was cribra orbitalia and cribra femoralis (12.%, 12/96). Finally, in 29 individuals (30.2%) there was no evidence of cribra

**
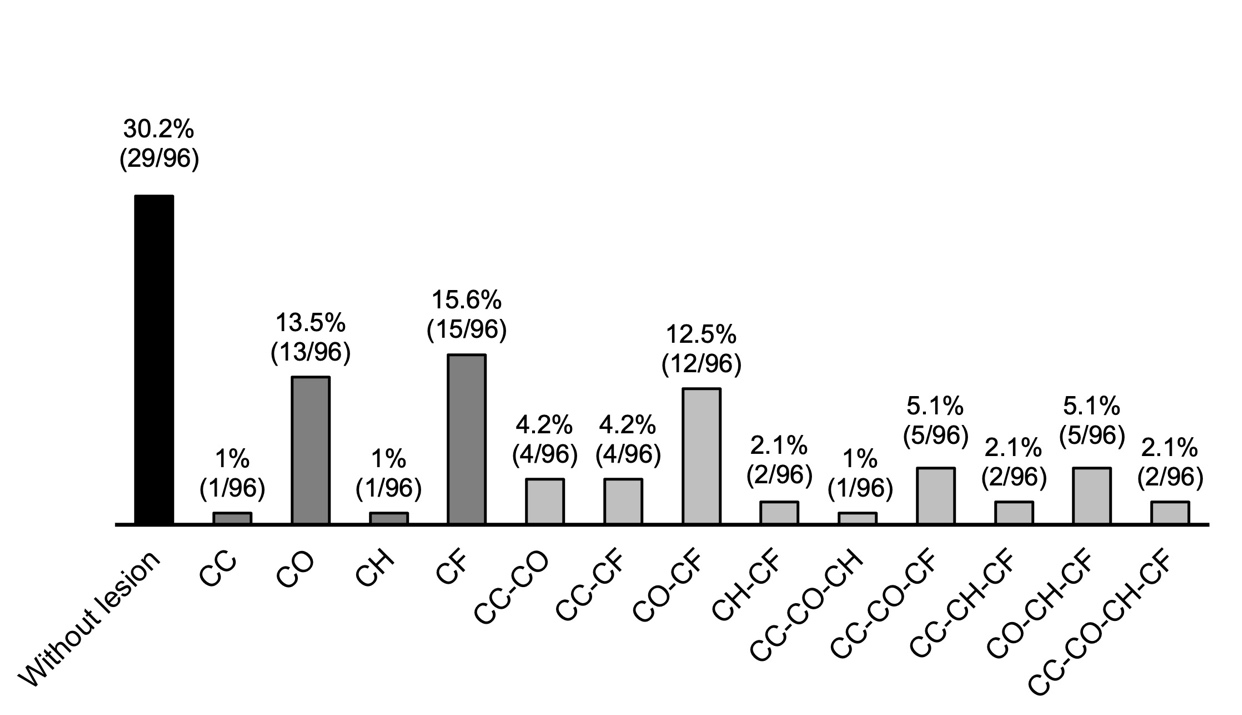
**

**Figure S1.** Frequencies of individuals (N=96) without cribra, with only one type of cribra and co-occurrence of cribra cranii (CC), cribra orbitalia (CO), cribra humeralis (CH), and cribra femoralis (CF). Only combinations with frequencies higher than zero are represented.

**Table S2.** Rotated (varimax) component matrix of the six elements and the Ca/P ratio with three extracted principal components and the total variance explained by each. In bold are the largest values for each component.

| **Element** | **Pc1** (48.5%) | **Pc2** (18.4%) | **Pc3** (13.9%) |
| --- | --- | --- | --- |
| **Sr** | **0.89** | -0.13 | -0.04 |
| **P** | **0.87** | 0.42 | -0.06 |
| **Ca** | **0.85** | 0.40 | -0.13 |
| **Ca/P** | **-0.55** | -0.32 | -0.37 |
| **S** | 0.10 | **0.92** | -0.03 |
| **Fe** | -0.23 | **-0.80** | -0.13 |
| **Pb** | -0.09 | 0.03 | **0.95** |

**Table S3.** Results of the logistic regression. Independent variables - age at death, PC1, and Pc2 - are the predictors for the occurrence (outcome) of cribra cranii, cribra orbitalia, cribra humeralis, and cribra femoralis. Statistically significant values (p<0.05) are in bold.

| **Porous skeletal lesions** | **Independent variables** | **B** | **SE** | **Wald** | ***Sig.*** | **Odds ratio** | **95% confidence interval** | |
| --- | --- | --- | --- | --- | --- | --- | --- | --- |
|  |  |  |  |  |  |  | **Lower** | **Upper** |
|  | | | | | | | | |
| Cribra cranii | Age at death | 0.07 | 0.39 | 0.31 | 0.08 | 1.00 | 0.92 | 4.34 |
|  | Cp1 | 0.10 | 0.29 | 0.11 | 0.74 | 1.10 | 0.62 | 1.97 |
|  | Cp2 | 0.03 | 0.34 | 0.01 | 0.92 | 1.04 | 0.53 | 2.03 |
|  | Cp3 | 0.02 | 0.27 | 0.01 | 0.94 | 1.02 | 0.60 | 1.74 |
|  | Constant | -2.70 | 0.84 | 10.33 | 0.001 | 0.07 | - | - |
|  | | | | | | | | |
| Cribra orbitalia | **Age at death** | **0.62** | **0.35** | **3.18** | **0.04** | **1.86** | **0.94** | **3.68** |
|  | Cp1 | 0.05 | 0.27 | 0.04 | 0.85 | 1.05 | 0.62 | 1.79 |
|  | **Cp2** | **0.57** | **0.31** | **3.44** | **0.03** | **1.76** | **0.97** | **3.20** |
|  | Cp3 | 0.25 | 0.26 | 0.93 | 0.33 | 1.20 | 0.77 | 2.14 |
|  | Constant | -0.87 | 0.66 | 1.75 | 0.17 | 0.42 | - | - |
|  |  |  |  |  |  |  |  |  |
| Cribra humeralis | **Age at death** | **2.12** | **0.57** | **13.67** | **<0.001** | **8.32** | **2.71** | **25.60** |
|  | Cp1 | 0.10 | 0.34 | 0.08 | 0.78 | 1.10 | 0.56 | 2.16 |
|  | Cp2 | -0.42 | 0.44 | 0.90 | 0.34 | 0.66 | 0.28 | 1.57 |
|  | Cp3 | -0.13 | 0.36 | 0.12 | 0.73 | 0.88 | 0.43 | 1.793 |
|  | Constant | -6.19 | 1.42 | 19.11 | <0.001 | 0.002 | - | - |
|  | | | | | | | | |
| Cribra femoralis | **Age at death** | **1.94** | **0.47** | **17.17** | **<0.001** | **6.97** | **2.78** | **17.45** |
|  | **Cp1** | **0.83** | **0.32** | **6.86** | **0.009** | **2.30** | **1.23** | **4.29** |
|  | **Cp2** | **0.35** | **0.34** | **1.08** | **0.04** | **1.42** | **0.73** | **2.74** |
|  | Cp3 | -0.15 | 0.30 | 0.25 | 0.62 | 0.86 | 0.48 | 1.55 |
|  | Constant | -3.39 | 0.82 | 16.94 | <0.001 | 0.03 | - | - |
|  |  |  |  |  |  |  |  |  |

**Supplementary text 2**

When analyzing the presence or absence of lesions, a significant association was observed with the groups of age at death (χ2=21.0, p<0.001). Thus, in this case, individuals without cribra were observed at a statistically significantly higher frequency in the group aged less than two years (infancy). Moreover, the frequency of individuals with the four types of cribra increased with age at death (Table S4). Yet, statistically significant associations were only observed for cribra humeralis and cribra femoralis (Table S4).

**Table S4.** Distribution of the groups of age at death according to different types of cribra. In bold are statistically significant (significance set at p<0.05).

| **Age groups** | **N** | **Cribra cranii** | | | |  | **Cribra orbitalia** | | | |  | **Cribra humeralis** | | | |  | **Cribra femoralis** | | | |
| --- | --- | --- | --- | --- | --- | --- | --- | --- | --- | --- | --- | --- | --- | --- | --- | --- | --- | --- | --- | --- |
|  |  | n | % | χ2 | Sig. |  | n | % | χ2 | Sig. |  | n | % | χ2 | Sig. |  | n | % | χ2 | Sig. |
| Infancy  (birth-2y) | 37 | 4 | 10.8 | 4.6 | 0.10 |  | 12 | 32.4 | 4.8 | 0.09 |  | 0 | - | 19.9 | **<0.001** |  | 7 | 18.9 | 32.4 | **<0.001** |
| Early childhood  (3-5 y) | 40 | 8 | 20.0 |  |  |  | 20 | 50.0 |  |  |  | 6 | 15.0 |  |  |  | 23 | 57.5 |  |  |
| Middle childhood  (6-11 y) | 19 | 7 | 36.8 |  |  |  | 10 | 52.6 |  |  |  | 7 | 36.8 |  |  |  | 16 | 84.2 |  |  |
| All | 96 | 19 | 19.8 | - | - |  | 42 | 43.8 | - | - |  | 13 | 13.5 | - | - |  | 46 | 47.9 | - | - |
